# Simultaneous Electrochemical Formation of Porous Silicon and Noble NPs for Au Nucleation Sites in SERS Substrates

**DOI:** 10.1021/acsmaterialsau.5c00169

**Published:** 2025-12-23

**Authors:** Chia-Chi Lu, Hsiao-Han Hsu, I-An Lin, Vincent K. S. Hsiao, Chih-Chien Chu

**Affiliations:** † Department of Medical Applied Chemistry, 34899 Chung Shan Medical University, Taichung 40201, Taiwan; ‡ Department of Applied Materials and Optoelectronic Engineering, 59433 National Chi Nan University, Nantou 545301, Taiwan; § Department of Medical Education, Chung Shan Medical University Hospital, Taichung 40201, Taiwan

**Keywords:** porous silicon, surface-enhanced Raman scattering (SERS), electrochemical etching, bimetallic interface, noble metal NPs

## Abstract

This study presents a one-step electrochemical etching strategy that enables the simultaneous formation of porous silicon (PSi) and in situ deposition of noble metal nanoparticles (NPs), which subsequently act as nucleation sites for gold growth to form bimetallic SERS substrates. Three metal precursors—K_2_PtCl_4_, HAuCl_4_, and K_2_PdCl_4_—were systematically compared to elucidate how precursor type affects PSi morphology, nanoparticle distribution, and subsequent gold deposition. SEM and EDS analyses revealed distinct deposition behaviors among the metals, leading to varied gold nucleation efficiencies and SERS enhancement levels. HAuCl_4_-treated substrates produced the highest absolute Raman intensity, while K_2_PtCl_4_-treated substrates exhibited lower intensity but superior spectral quality with minimal fluorescence background and sharper peaks. XRD and XPS confirmed successful gold deposition and precursor-dependent interfacial interactions, including the formation of Pt–Au bimetallic interfaces. Reusability tests demonstrated that the Pt-assisted substrates maintained stable performance over multiple cycles, confirming their structural robustness and practical feasibility. Overall, this work provides mechanistic insight into how noble metal interfaces govern SERS spectral characteristics and establishes a rational pathway for designing PSi-based SERS substrates emphasizing spectral precision, reproducibility, and reusability rather than mere sensitivity enhancement.

## Graphical abstract

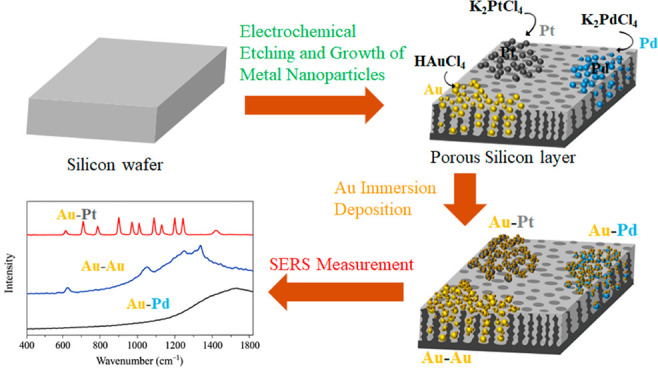

## 1 Introduction

Surface-enhanced Raman scattering (SERS) spectroscopy is a powerful vibrational technique that enables ultrasensitive chemical identification of low-concentration analytes through localized surface plasmon resonance (LSPR)–induced electromagnetic field amplification.^1–4^ Owing to its remarkable signal enhancement, SERS has been widely applied in sensing, single-molecule spectroscopy, biomedical diagnostics, and photocatalysis.^5–7^ Compared with conventional Raman spectroscopy, which is limited by weak molecular scattering cross sections, SERS can achieve single-molecule detection under optimized conditions. The SERS enhancement originates from both electromagnetic and chemical contributions.^8,9^ The electromagnetic mechanism dominates, arising from the intense localized fields generated when incident light resonates with surface plasmons of noble-metal nanostructures. Under resonance conditions, “hot spots” form at nanoscale gaps, tips, or rough regions.^10^ Chemical enhancement, though typically weaker, involves charge-transfer interactions between molecules and metal surfaces.^11^ Because SERS performance relies heavily on such localized hot spots, numerous chemical and physical strategies have been developed to create dense plasmonic junctions and improve sensitivity.^12–15^ Physical enrichment methods—such as employing superhydrophobic surfaces or exploiting the coffee-ring effect—are particularly attractive for their label-free and reaction-free nature.^16,17^ Conventional SERS substrates primarily use silver or gold because of their strong plasmonic responses in the visible–near-infrared region. However, achieving high reproducibility, strong enhancement factors, and low cost simultaneously remains challenging. Three-dimensional (3D) hierarchical SERS substrates have thus received increasing attention.^18,19^ Compared with 2D or colloidal systems, 3D architectures extend plasmonic active sites along the *z*-axis, improving both sensitivity and reproducibility, and they can often be reused after photocatalytic cleaning. Reported 3D SERS platforms include porous graphene foams,^20^ metal foams,^21^ gold aerogels,^22^ and ordered Au nanoframe structures.^23^ While these systems show excellent performance, their fabrication processes are typically complex and expensive.

Porous silicon (PSi) offers a versatile template for SERS owing to its high surface-to-volume ratio, tunable porosity, biocompatibility, and compatibility with semiconductor processing.^24–26^ Because silicon lacks intrinsic plasmonic activity, noble-metal deposition is required to achieve enhancement.^27–34^ Among various fabrication routes, metal-assisted chemical etching (MACE) provides a simple, low-cost, and scalable approach to produce nanostructured silicon without external bias. Recent reports have demonstrated Au-NP-embedded 3D PSi scaffolds with enhancement factors up to 10^9^, enabling picomolar detection of dye molecules.^35^ Electrochemical etching combined with metal deposition offers another controllable route for metallized PSi. In typical two-step methods, PSi is first prepared and then metallized via solution-based or vapor-deposition processes.^25,36^ While vapor techniques yield uniform films, they demand costly vacuum systems and often produce overly smooth surfaces lacking sufficient roughness for hot-spot generation. Bimetallic nanostructures^37^ have recently attracted interest for their synergistic catalytic and plasmonic properties. Tunable composition and interfacial coupling enable improved electromagnetic and charge-transfer effects compared with monometallic systems.

The present study aims to develop an one-step electrochemical etching strategy for fabricating noble-metal-modified PSi SERS substrates. Three noble-metal precursors—K_2_PtCl_4_, HAuCl_4_, and K_2_PdCl_4_—were systematically investigated to compare their behaviors during etching and their roles as nucleation sites for subsequent gold growth. By directly introducing these precursors into the etching solution, simultaneous PSi formation and in situ noble-metal nanoparticle generation were achieved on low-resistivity Si within ≈3 min. The process relies on controllable redox reactions between metal ions and the Si surface, enabling uniform metal nucleation without additional reducing agents or contamination. Afterward, gold deposition on the in situ metal seeds produced Au–M (M = Pt, Pd, Au) bimetallic nanostructures with tunable plasmonic properties. Systematic SERS evaluation using rhodamine 6G revealed that HAuCl_4_-derived samples provided the strongest absolute enhancement, K_2_PtCl_4_-derived samples achieved the highest signal-to-noise ratio with minimal fluorescence background, and K_2_PdCl_4_-derived samples showed weaker enhancement. This facile process eliminates complex pretreatments and multistep reductions, offering rapid, reproducible, and low-cost fabrication of PSi-based bimetallic SERS substrates and new insights into noble-metal roles in plasmonic coupling.

## 2 Experimental Section

Low-resistivity p-type silicon wafers (0.001–0.004 Ω·cm) were used as substrates. Noble metal precursors included potassium tetrachloroplatinate (II) (K_2_PtCl_4_, ≥99%, ACROS), gold­(III) chloride trihydrate (HAuCl_4_·3H_2_O, ≥99%, ACROS), and potassium­(II) tetrachloropalladate (K_2_PdCl_4_, ≥99%, ACROS). Hydrofluoric acid (HF, 49%, analytical grade), anhydrous ethanol (C_2_H_5_OH, ≥99.5%, analytical grade), and deionized (DI) water were used to prepare the etching solutions. Rhodamine 6G (R6G, Sigma-Aldrich) served as the probe molecule for SERS measurements. All chemicals were used as received without further purification. Silicon wafers were cut into 1.5 cm × 1.5 cm squares and sequentially cleaned by ultrasonication in acetone, isopropanol, and DI water (10 min each) to remove organic residues and particulates. The substrates were dried under nitrogen and immediately subjected to electrochemical etching. A two-electrode configuration was employed, with the silicon substrate as the working electrode and a platinum foil (2 cm × 5 cm) as the counter electrode.^38^ The etching solution was prepared by mixing the metal precursor solution, ethanol, and HF in a 1:1:1 volume ratio. Precursor concentrations of 0.5, 1, 2, and 5 mM were tested, while control samples were prepared by replacing the precursor solution with DI water. Electrochemical etching was performed at room temperature using a source meter under a constant current density of 40 mA/cm^2^ for 3 min. After etching, the substrates were rinsed with DI water and dried under nitrogen. Gold nanoparticle deposition was carried out via a modified seed-mediated growth method. The deposition solution consisted of 1 mL of 5 mM HAuCl_4_, 4 mL of 0.21 mM methyl orange, and 0.5 mL of 100 mM sodium citrate. Etched Si substrates were gently rinsed with DI water and ethanol to remove residual etching solution, followed by immediate immersion into the deposition solution. The reaction proceeded at room temperature for 2 h under static conditions to allow sufficient Au nucleation and growth. Upon completion, the substrates were rinsed thoroughly with DI water until the rinsing solution became colorless, followed by ethanol washing and drying under nitrogen at room temperature. The resulting substrates were used immediately for surface characterization and SERS measurements. SERS spectra were collected on a micro-Raman spectrometer (Horiba HR-320) using a 633 nm He–Ne laser (5 mW) with a 40× objective lens (laser spot size of 2 μm, estimated laser intensity of 10^5^ W/cm^2^), an integration time of 10 s, and a grating of 300 lines/mm.^39^ Reusability was evaluated using substrates etched with 1 mM K_2_PtCl_4_ precursor. After each measurement, the substrate was immersed in ethanol for 10 min to remove adsorbed probe molecules, rinsed with DI water, and dried under nitrogen. This cleaning–measurement cycle was repeated 10 times to assess the substrate’s reusability.

## 3 Results and Discussion

### 3.1 Influence of Different Metal Precursors on PSi Formation and Gold Nanoparticle Generation

Figure 1 compares the surface morphologies of PSi etched with different metal precursors before gold deposition. The DI water control (Figure 1a) shows a basic porous network representing pure silicon dissolution. In contrast, the K_2_PtCl_4_-etched sample (Figure 1b) displays uniformly dispersed bright dots identified as Pt nanoparticles, nanometers in size, confirming in situ Pt deposition with well-controlled nucleation during etching. The HAuCl_4_- (Figure 1c) and K_2_PdCl_4_-etched (Figure 1d) samples exhibit modified surface textures, though Au and Pd particles are not clearly resolved at this scale. Their presence is confirmed by XPS and indirectly supported by enhanced SERS activity after gold deposition. The distinct visibility of Pt arises from its discrete, low-density nanoparticles, whereas Au and Pd likely form finer dispersions blending into the porous matrix. Collectively, the morphological variations and compositional analyses demonstrate that each precursor alters the etching dynamics and enables in situ metal nucleation, establishing active sites for subsequent gold growth.

**Figure 1:**
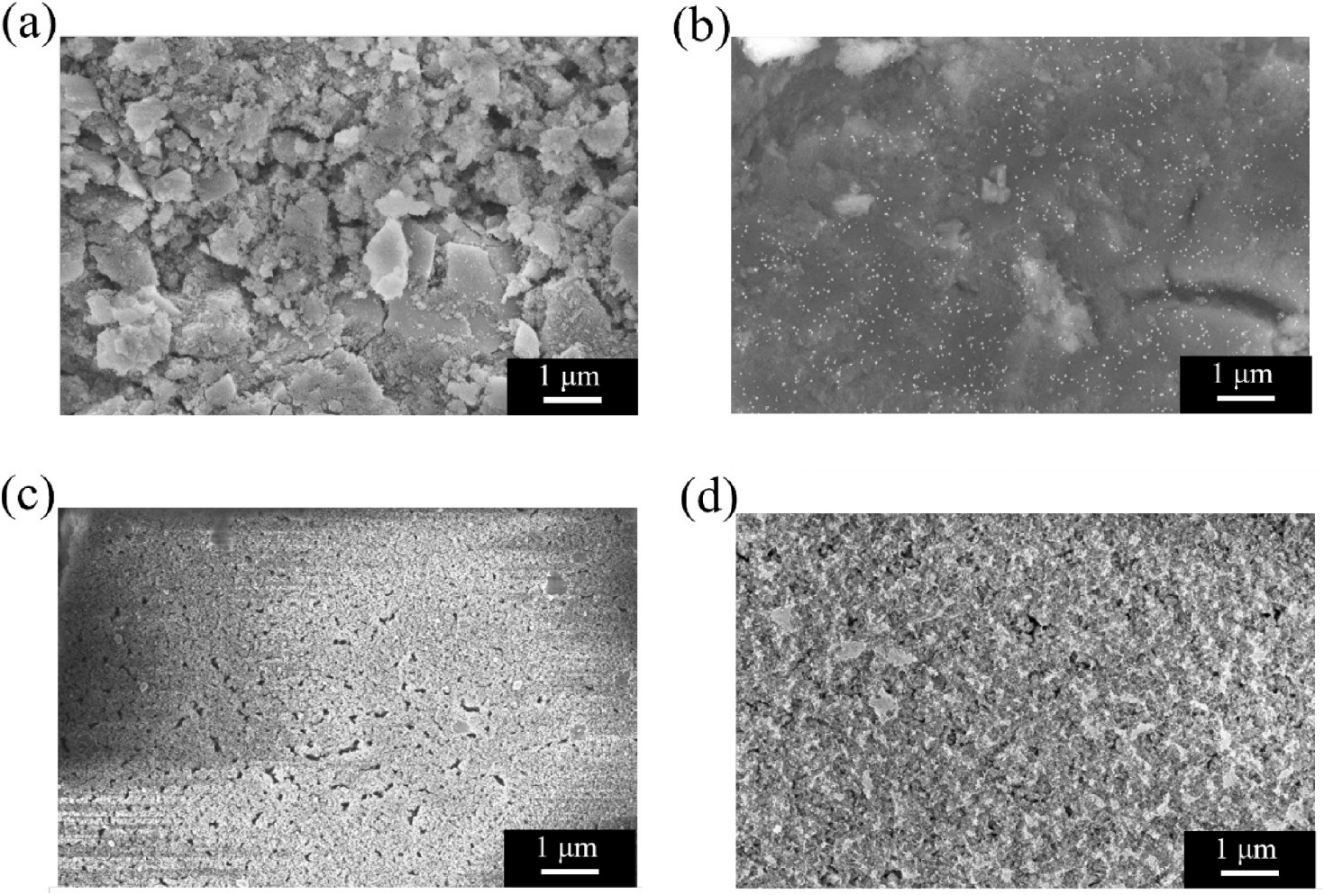
SEM images of PSi surfaces after electrochemical etching with different metal precursors (1 mM) prior to gold deposition: (a) DI water control showing a basic porous morphology; (b) K_2_PtCl_4_-etched surface exhibiting uniformly dispersed Pt nanoparticles (bright dots) across the porous matrix; (c) HAuCl_4_-etched surface with modified pore structure; and (d) K_2_PdCl_4_-etched surface with altered surface texture.

To further verify the in situ metal deposition during electrochemical etching, elemental mapping and EDS analyses were performed on PSi surfaces after subsequent gold deposition (Figure 2). The DI-water–etched control (Figure 2a) shows an irregular porous morphology with discontinuous Au coverage. The EDS mapping reveals dominant Si signals with sparse Au distribution, consistent with incomplete or island-like gold growth on the unmodified surface. Quantitatively, the EDS spectrum indicates 72.2 wt % Si and 24.7 wt % Au, corresponding to a limited Au atomic fraction of only 4.6%. In contrast, the K_2_PtCl_4_-etched sample (Figure 2b) exhibits a more uniform and continuous Au layer. The elemental maps show intensive Au and Pt signals evenly spread across the porous matrix, indicating that Pt nanoparticles act as efficient nucleation centers for subsequent gold deposition. The corresponding EDS spectrum reveals 91.2 wt % Au with an atomic fraction of 74.6%, confirming substantially improved Au coverage compared with the control. This enhancement supports the catalytic role of predeposited Pt in facilitating gold reduction and promoting uniform film growth during the postdeposition process. These compositional results corroborate the SEM observations in Figure 1 where Pt-assisted etching not only modifies the surface morphology but also introduces active metallic sites that accelerate gold nucleation, leading to denser and more homogeneous Au coverage on the porous silicon framework.

**Figure 2:**
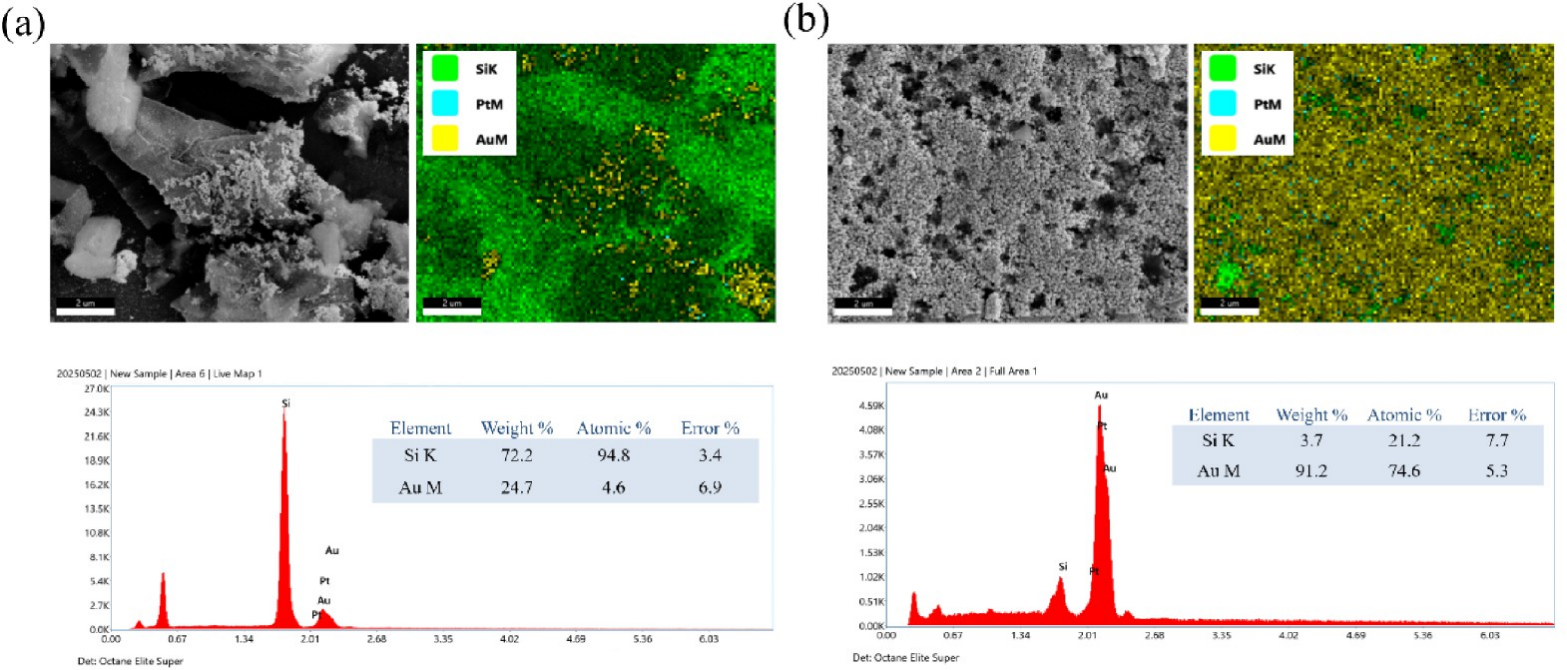
SEM–EDS analyses of PSi surfaces after gold deposition: (a) DI-water–etched control showing discontinuous Au coverage with sparse elemental distribution; (b) K_2_PtCl_4_-etched sample exhibiting uniform and dense Au deposition aided by Pt nucleation sites.

To further compare the influence of Au- and Pd-based precursors on subsequent gold growth, SEM–EDS analyses were conducted on PSi surfaces after gold deposition (Figure 3). The sample pre-etched in 1 mM HAuCl_4_ (Figure 3a) shows a relatively smooth and partially covered surface. Elemental mapping reveals scattered Au signals distributed across the Si framework, with minor local enrichment. The EDS spectrum indicates 6.8 wt % Au (9.7 at%), suggesting moderate Au deposition efficiency, consistent with a limited number of preexisting Au nucleation sites formed during etching. In contrast, the K_2_PdCl_4_-etched sample (Figure 3b) exhibits a denser and more textured Au layer. The elemental maps show strong Au and Pd signals that coincide spatially, indicating that Pd nanoparticles effectively serve as heterogeneous nucleation centers to catalyze Au reduction. Quantitative EDS analysis reveals 10.8 wt % Au (14.8 at%), notably higher than in the HAuCl_4_-etched sample. This confirms that Pd-assisted etching enhances the subsequent Au deposition, yielding thicker and more continuous coverage. These results demonstrate that both Au- and Pd-precursor treatments modify the PSi surface chemistry and electrochemical reactivity, but Pd provides a stronger catalytic effect on gold growth. Together with the Pt-assisted case (Figure 2), these findings highlight the role of noble-metal seeds formed during etching in promoting uniform and extensive Au deposition on porous silicon.

**Figure 3:**
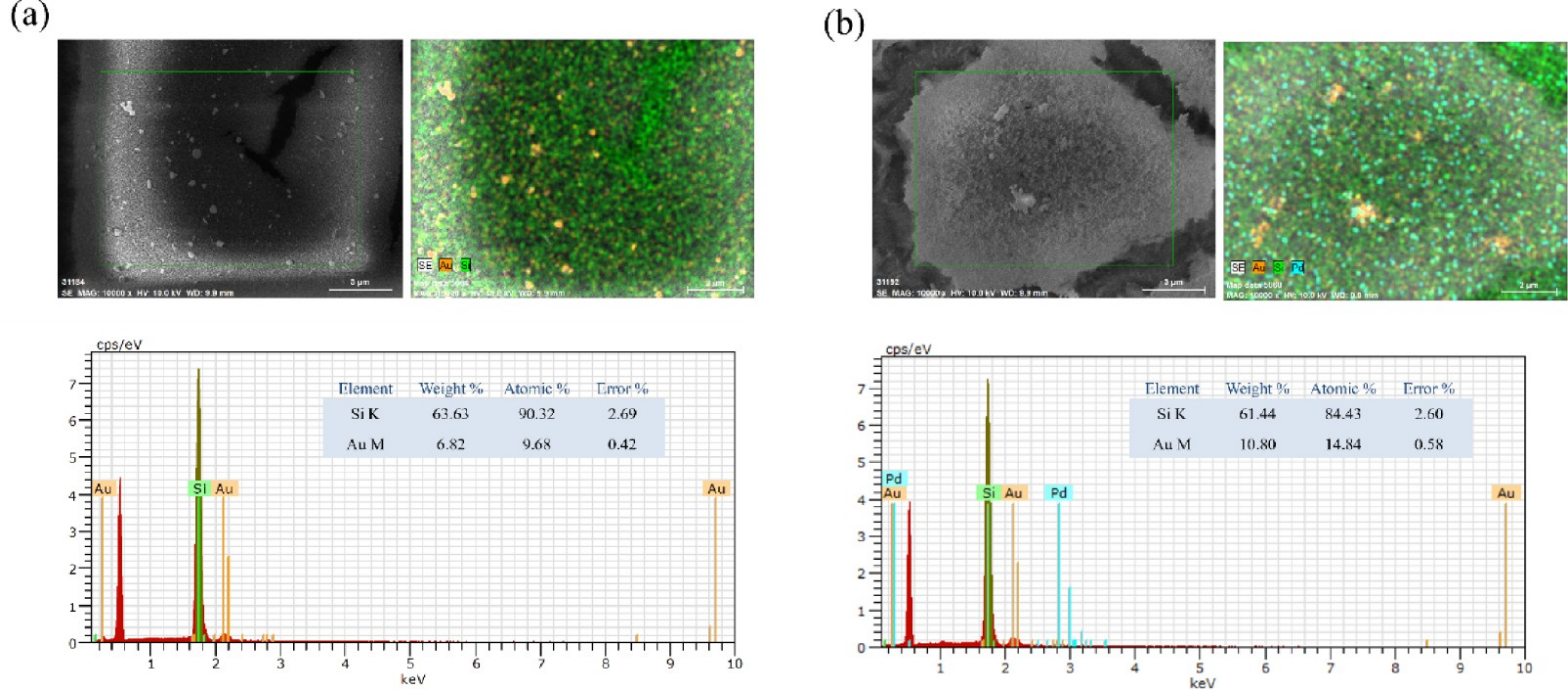
SEM–EDS analyses of PSi surfaces after gold deposition following etching with (a) 1 mM HAuCl_4_ and (b) 1 mM K_2_PdCl_4_.

To provide further evidence for the role of in situ metal nanoparticles in directing gold growth, cross-sectional SEM–EDS analyses were performed on PSi samples after gold deposition (Figure 4). The DI-water–etched control (Figure 4a) shows a relatively sparse and discontinuous Au distribution along the porous layer. Elemental mapping indicates that Au is scattered mainly near the top surface with limited penetration into the porous framework, consistent with inefficient nucleation and island-like deposition. The EDS quantification (13.7 wt % Au, 2.3 at%) confirms the low Au loading, which correlates with poor SERS enhancement due to the absence of dense plasmonic junctions. In contrast, the K_2_PtCl_4_-etched sample (Figure 4b) exhibits a highly integrated Au–Pt–Si composite structure. The cross-sectional maps clearly show overlapping Pt and Au signals distributed throughout the upper porous region, revealing that Pt nanoparticles act as nucleation centers for Au deposition and promote vertical growth within the pore network. The higher Au content (26.2 wt %, 8.8 at%) confirms a more efficient and continuous gold coating. The spatial colocalization of Pt and Au suggests that the Pt nanoparticles not only facilitate Au reduction but also generate abundant nanoscale junctions that serve as SERS “hot spots,” significantly enhancing electromagnetic coupling. These cross-sectional results complement the surface analyses, collectively confirming that Pt-assisted electrochemical etching establishes effective nucleation templates for gold growth, leading to denser metallic connectivity and improved SERS performance compared with the DI-water control.

**Figure 4:**
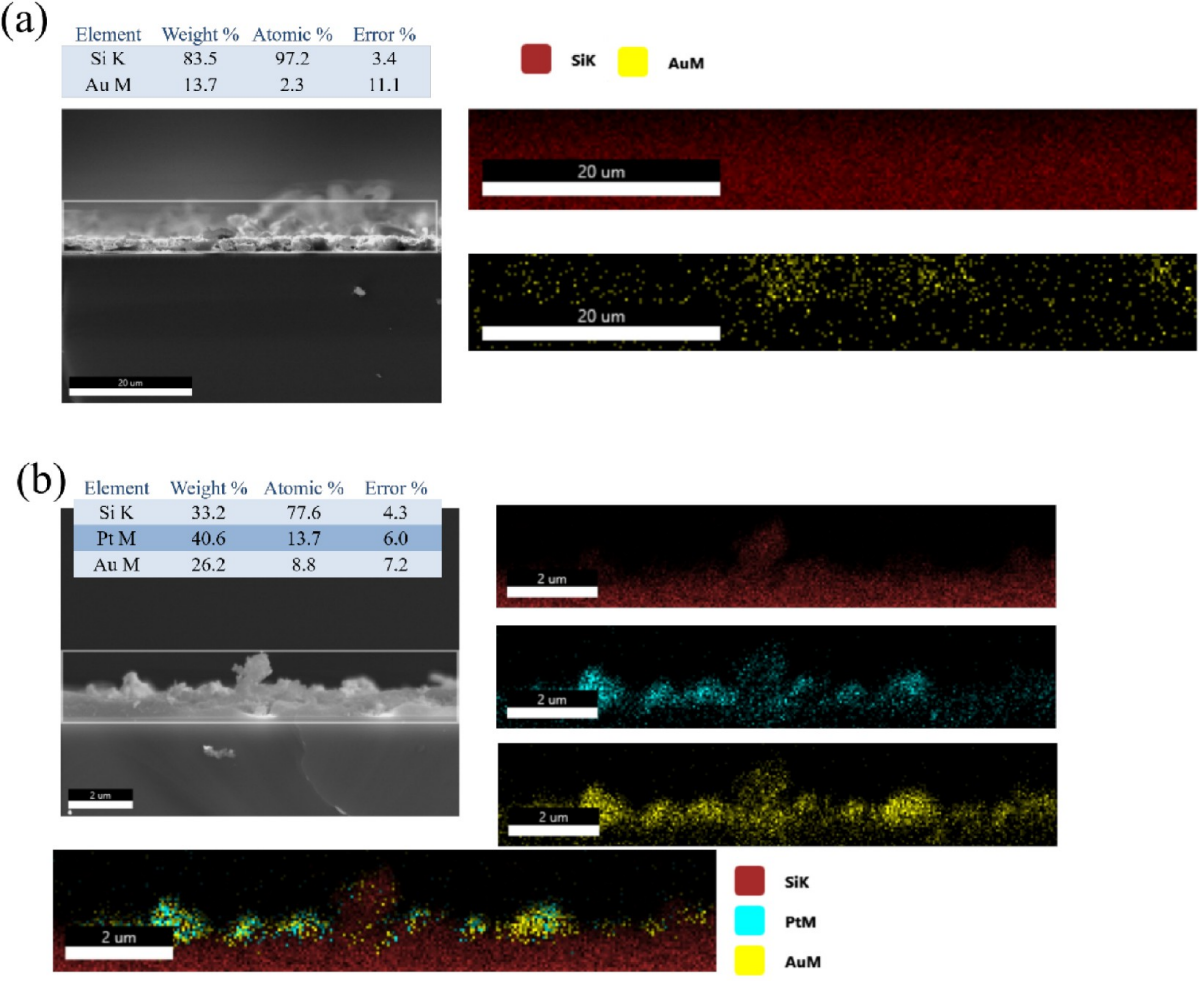
Cross-sectional SEM–EDS analyses of (a) DI-water–etched and (b) K_2_PtCl_4_-etched PSi after gold deposition. The overlapping Pt and Au signals confirm that Pt nanoparticles serve as nucleation sites for gold growth and form abundant SERS hot spots within the porous framework.

### 3.2 SERS Spectral Performance Analysis

Figure 5 compares the Raman spectra of R6G collected on PSi substrates treated with different metal precursors, before and after gold immersion deposition. Prior to deposition, the K_2_PdCl_4_-treated sample exhibited the strongest signal near 520 cm^–1^, mainly originating from the intrinsic Raman scattering of the silicon substrate rather than from R6G molecules.^40^ The HAuCl_4_-treated surface produced a moderate response, the K_2_PtCl_4_-treated one showed the weakest, and the DI-water control yielded almost no detectable signal. These observations agree with the SEM results, confirming that distinct metal precursors alter the surface chemistry and nucleation behavior during etching. After gold deposition, all samples displayed clear SERS enhancement, but with different magnitudes and spectral qualities. The HAuCl_4_-treated substrate exhibited the highest absolute intensity (∼6000 au) and an SNR of approximately 35, with sharp and well-resolved R6G peaks at 610, 770, 1310, 1360, 1510, and 1570 cm^–1^.^41^ The K_2_PdCl_4_-treated sample reached a maximum intensity of ∼7000 au, yet its baseline remained high (SNR ≈ 18) due to strong fluorescence interference, indicating that its initial intensity mainly originated from Si Raman scattering rather than true SERS enhancement. In comparison, the K_2_PtCl_4_-treated substrate showed lower absolute intensity (∼400 au) but achieved the highest relative enhancement (∼8-fold) and the best SNR ≈ 42, benefiting from a much lower fluorescence background. The reduced fluorescence of the K_2_PtCl_4_-derived substrate yields sharper and more symmetric R6G bands, suggesting that Pt–Au heterointerfaces produce a more homogeneous local electromagnetic field and minimize variations among enhancement sites.^42–44^ The Pt nanoparticles also promote more uniform adsorption configurations, leading to less conformational heterogeneity and suppressed peak broadening. Moreover, Pt’s higher work function and distinct d-orbital structure may facilitate interfacial charge transfer with Au, producing localized yet uniform enhancement fields.^45^ Such effects result in improved spectral reproducibility and signal-to-noise characteristics, crucial for trace-level SERS analysis under long integration times. By contrast, the HAuCl_4_-treated substrate benefited from dense Au nucleation and Au–Au coupling, while the K_2_PdCl_4_ sample suffered from fluorescence background and limited SERS activity. The DI-water control remained essentially inactive even after gold deposition, confirming that metal-ion preconditioning is essential for establishing SERS-active interfaces.

**Figure 5:**
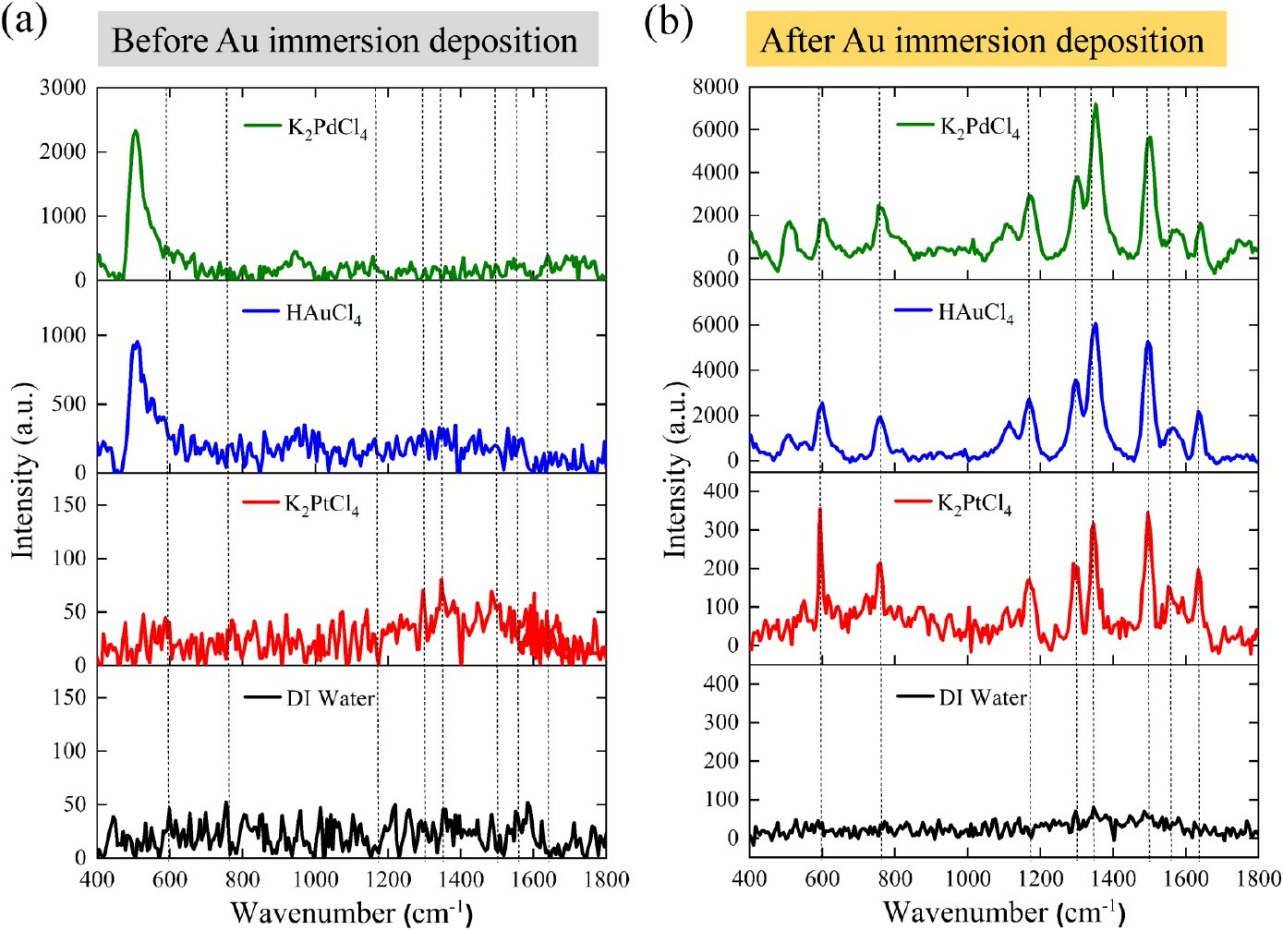
Raman spectra of 10^–5^ M R6G on PSi substrates etched with different 1 mM metal precursors (K_2_PdCl_4_, HAuCl_4_, K_2_PtCl_4_, and DI water): (a) before and (b) after gold immersion deposition. After deposition, the HAuCl_4_-treated sample exhibits the highest absolute intensity (∼6000 au, SNR ≈ 35), the K_2_PdCl_4_-treated sample shows strong but fluorescence-contaminated signals (∼7000 au, SNR ≈ 18), and the K_2_PtCl_4_-treated sample demonstrates a lower intensity (∼400 au) yet the best SNR ≈ 42 owing to minimal background fluorescence. Characteristic R6G bands appear at 610, 770, 1310, 1360, 1510, and 1570 cm^–1^.

Although the absolute Raman intensity of the K_2_PtCl_4_-treated sample is lower than that of the HAuCl_4_- and K_2_PdCl_4_-treated substrates, this characteristic can be advantageous for certain sensing scenarios. In practical SERS measurements, signal amplification can be achieved by extending the integration or accumulation time; however, excessive fluorescence background often limits this approach by saturating the detector and deteriorating the spectral baseline. The significantly lower fluorescence background in Pt-treated samples can be attributed to the distinct porous silicon morphology formed during electrochemical etching with different metal precursors. As shown in the SEM-EDS characterization, the Pt-precursor etching process produces a unique pore structure and nanoparticle distribution pattern. Different pore sizes, surface roughness, and nanoparticle arrangements influence the interaction between the excitation laser and the substrate, thereby affecting background fluorescence levels. The porous silicon substrate itself can contribute to fluorescence, and this contribution appears to be minimized in the Pt-etched structures, possibly due to reduced light scattering within the porous network or different surface oxidation states. The Pt-assisted substrate, with its intrinsically low background emission, allows longer integration times without introducing significant noise accumulation. As a result, the weaker initial signal can be compensated through time-dependent averaging, ultimately achieving high detection sensitivity with improved spectral clarity. Such low-background substrates are particularly valuable for trace-level or slow-kinetic analyses, where extended acquisition is required to enhance signal strength while maintaining spectral fidelity. Therefore, despite its lower initial intensity, the K_2_PtCl_4_-derived sample offers practical benefits for long-term accumulation measurements, enabling reliable detection of weak Raman signals that would otherwise be obscured on fluorescence-dominated substrates.

### 3.3 XRD Structural Analysis

Figure 6 presents XRD patterns of samples treated with different metal precursors after gold deposition. The full-range patterns (Figure 6a) confirm successful Au deposition on all substrates, with characteristic Au diffraction peaks at 38.2° (111), 44.4° (200), 64.6° (220), and 77.5° (311) corresponding to the face-centered cubic structure of gold.^46^ Silicon substrate peaks at 28.4° (111) and 56.1° (311) are only observed in the K_2_PdCl_4_, confirming preservation of the underlying PSi structure. In contrast, HAuCl_4_- and K_2_PtCl_4_-treated samples display well-defined Au peaks with reduced Si substrate signals, indicating more effective gold coverage. This result confirms that both Au and Pt nanoparticles serve as effective nucleation sites for subsequent Au growth, with Pt proving particularly efficient despite its lower initial particle density. It should be noted that the relative intensity of Si substrate peaks varies across different samples, reflecting differences in Au surface coverage. The more prominent Si peaks in K_2_PdCl_4_-treated samples are consistent with their lower effective Au coverage due to the aggregated Pd morphology limiting uniform Au deposition. Figure 6b shows a magnified view of the Au (111) peak region, revealing subtle but important differences in peak characteristics among the samples. The K_2_PtCl_4_-treated sample exhibits a noticeably narrower peak with a smaller full width at half-maximum (fwhm) compared to HAuCl_4_- and K_2_PdCl_4_-treated samples. According to the Scherrer equation, peak narrowing indicates larger crystallite size or reduced lattice strain in the deposited gold nanoparticles. This observation suggests that gold deposited on Pt nucleation sites possesses improved crystallinity, possibly due to the favorable lattice matching between Pt (a = 3.92 Å) and Au (a = 4.08 Å) and the formation of well-ordered Pt–Au bimetallic interfaces. The enhanced crystallinity may contribute to the superior spectral quality (sharper Raman peaks and reduced background) observed in SERS measurements for K_2_PtCl_4_-treated substrates, as more uniform crystal structures can generate more homogeneous electromagnetic field distributions.

**Figure 6:**
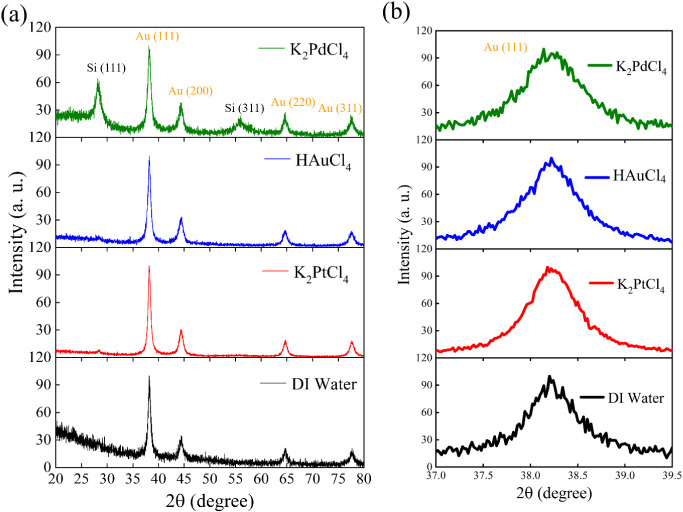
XRD patterns of PSi samples treated with different metal precursors after gold immersion deposition: (a) full-range patterns showing Au (111), (200), (220), and (311) peaks together with Si (111) and (311) reflections; (b) magnified view of the Au (111) region.

### 3.4 XPS Surface Chemical State Analysis

Figure 7 shows the XPS spectra of PSi samples treated with different metal precursors before and after Au deposition, focusing on the Si 2p, O 1s, and Au 4f regions. XPS analysis provides insights into the surface chemical environment and reaction mechanisms during electrochemical etching and subsequent Au deposition. In the Si 2p region (96–108 eV), all samples exhibited noticeable spectral changes after Au deposition. Before deposition, the Si 2p peak appeared at 103–104 eV, characteristic of SiO_2_, confirming partial surface oxidation.^47^ After Au deposition, peak broadening and intensity redistribution occurred, indicating altered surface chemical states. The K_2_PdCl_4_-treated sample showed only minor changes, likely due to Pd nanoparticle coverage that partially shielded the Si signal. In contrast, the HAuCl_4_-treated sample exhibited clear peak splitting, reflecting the coexistence of multiple silicon oxidation environments. The K_2_PtCl_4_-treated sample displayed the most significant broadening and uniform peak intensity, suggesting the formation of a distinct Pt–Au interfacial environment and modified surface oxidation state.^48^ Similar variations were observed in the O 1s region (528–538 eV). Initially centered near 533 eV (Si–O bonds),^48^ the O 1s peaks of metal-treated samples became asymmetric and shifted after Au deposition, indicating diverse oxygen chemical states arising from metal–oxide interactions. The K_2_PtCl_4_-treated sample showed the most pronounced asymmetry, implying the coexistence of multiple oxidation environments at the Pt–Au–Si interface. These results suggest that each metal precursor induces a distinct surface chemistry, which in turn influences SERS activity. The homogenized and electronically stable interface of the Pt–Au system likely contributes to sharper Raman peaks and reduced fluorescence background. The Au 4f spectra (80–92 eV) confirmed the successful formation of metallic Au NPs, with typical doublet peaks at ∼84 eV (Au 4f_7_/_2_) and ∼87.5 eV (Au 4f_5_/_2_), separated by ∼3.5 eV.^49^ The K_2_PdCl_4_-treated sample showed the strongest Au 4f intensity, consistent with its high Au loading. The HAuCl_4_-treated sample exhibited moderately intense, symmetric peaks, while the K_2_PtCl_4_-treated sample showed slightly weaker intensity but a narrower fwhm, indicating highly crystalline and chemically uniform Au NPs. A slight positive binding-energy shift of the Au 4f peaks for the K_2_PtCl_4_-treated sample suggests electronic coupling between Pt and Au, confirming the formation of genuine Pt–Au bimetallic interfaces rather than a simple physical mixture. These interfacial electronic interactions may underlie the superior SERS spectral sharpness and signal stability observed for the Pt-assisted substrate.

**Figure 7:**
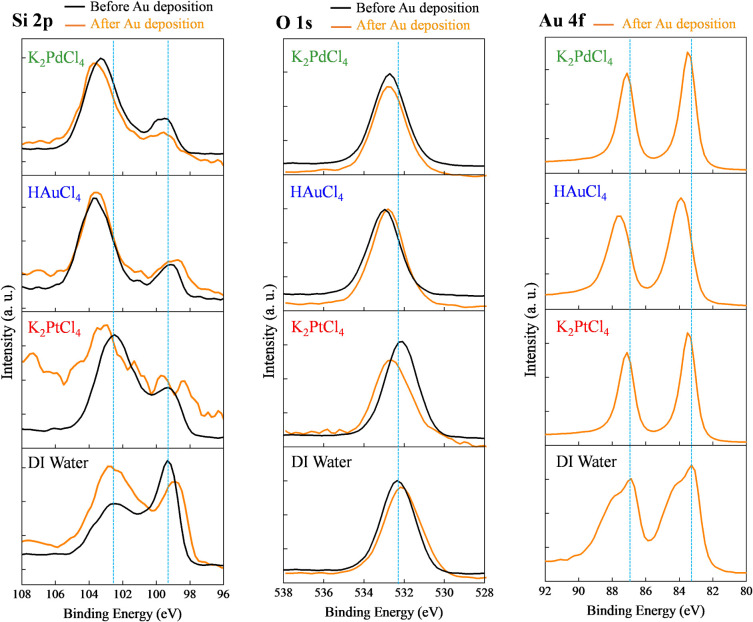
Comprehensive XPS analysis of samples treated with different metal precursors before and after Au deposition. From left to right: Si 2p region (96–108 eV), O 1s region (528–538 eV), and Au 4f region (80–92 eV). Black lines represent spectra before Au deposition; orange lines represent spectra after deposition. Blue dashed lines indicate the main peak positions.

### 3.5 Effect of Metal Precursor Concentration on PSi Formation and Gold Nanoparticle Growth

At 0.5 mM, the K_2_PtCl_4_-etched Si surface (Figure 8a) exhibits only a few discernible bright spots, indicating a low nucleation density of Pt nanoparticles. The sparse distribution suggests that Pt^4+^ reduction occurs only at limited active sites under these conditions, resulting in incomplete surface coverage. In comparison, the HAuCl_4_-etched surface (Figure 8b) shows higher particle density and a broader size range (15–50 nm), while the K_2_PdCl_4_-etched surface (Figure 8c) displays clustered regions with irregular aggregation. Increasing the precursor concentration to 5 mM amplified these differences. The K_2_PtCl_4_ system (Figure 8d) produced a markedly higher particle density with visible discrete nanoparticles (30–60 nm), reflecting enhanced but still well-controlled reduction kinetics. In the HAuCl_4_ system (Figure 8e), the surface evolved into a dense, near-continuous metallic layer, whereas the K_2_PdCl_4_ system (Figure 8f) showed extensive aggregation into a continuous film-like structure. These observations confirm that precursor concentration strongly influences nanoparticle formation behavior. The Pt precursor exhibits the slowest reduction rate, producing low nucleation at low concentration but stable particle growth at higher levels, while Au and Pd show more rapid and less controllable reduction.

**Figure 8:**
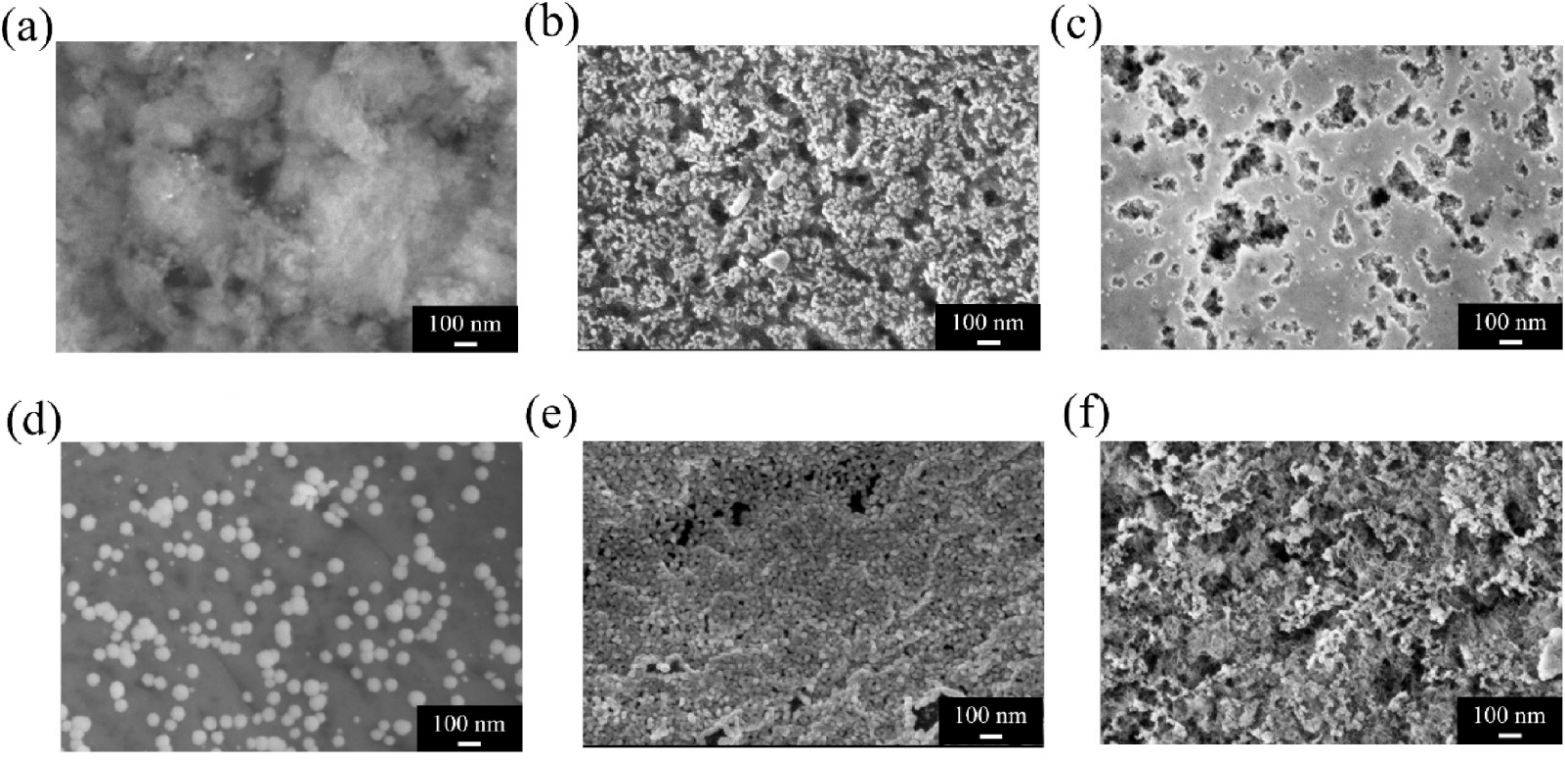
High-magnification SEM images of PSi surfaces etched with different metal precursors at (a–c) 0.5 mM and (d–f) 5 mM before gold deposition: (a, d) K_2_PtCl_4_; (b, e) HAuCl_4_; (c, f) K_2_PdCl_4_.

To evaluate the effectiveness of different metal precursors as nucleation sites for gold growth, SEM–EDS elemental mapping was performed on samples prepared with 0.5 mM precursors after gold immersion deposition. Figure 9 compares the spatial distribution of Au and Si elements with corresponding quantitative analyses. The K_2_PtCl_4_-pretreated sample (Figure 9a) shows nearly continuous Au coverage, with uniform yellow contrast and only weak Si signal in small void regions. EDS quantification indicates ∼76 at. % Au and ∼19 at. % Si, confirming extensive gold metallization even at this low precursor concentration. Despite the sparse Pt nanoparticle distribution before deposition (Figure 8a), these few Pt sites effectively catalyzed Au growth, consistent with the high interfacial activity of Pt toward Au reduction. In contrast, the HAuCl_4_-pretreated sample (Figure 9b) exhibits heterogeneous gold coverage with many Si-exposed areas. The Au content decreases to ∼13 at. %, while Si dominates the surface (87 at. %). The incomplete metallization suggests that low initial Au concentration provides insufficient nucleation density for uniform deposition. The K_2_PdCl_4_-pretreated sample (Figure 9c) shows the weakest Au signal (∼8 at. %) and intense, uniform Si mapping, indicating minimal gold deposition. Au appears mainly at isolated clusters, corresponding to aggregated Pd regions observed before gold growth (Figure 8c). These results demonstrate that Pd seeds, especially when clustered, do not efficiently promote Au nucleation. The EDS mapping highlights clear differences in gold deposition efficiency among precursors. Pt provides the most effective nucleation sites, achieving nearly full Au coverage from only 0.5 mM solution, whereas Au and Pd precursors require higher concentrations for comparable metallization. The superior performance of the Pt-assisted system underscores that precursor type and interfacial compatibility, rather than nanoparticle density alone, are the key factors governing effective bimetallic formation and subsequent SERS performance.

**Figure 9:**
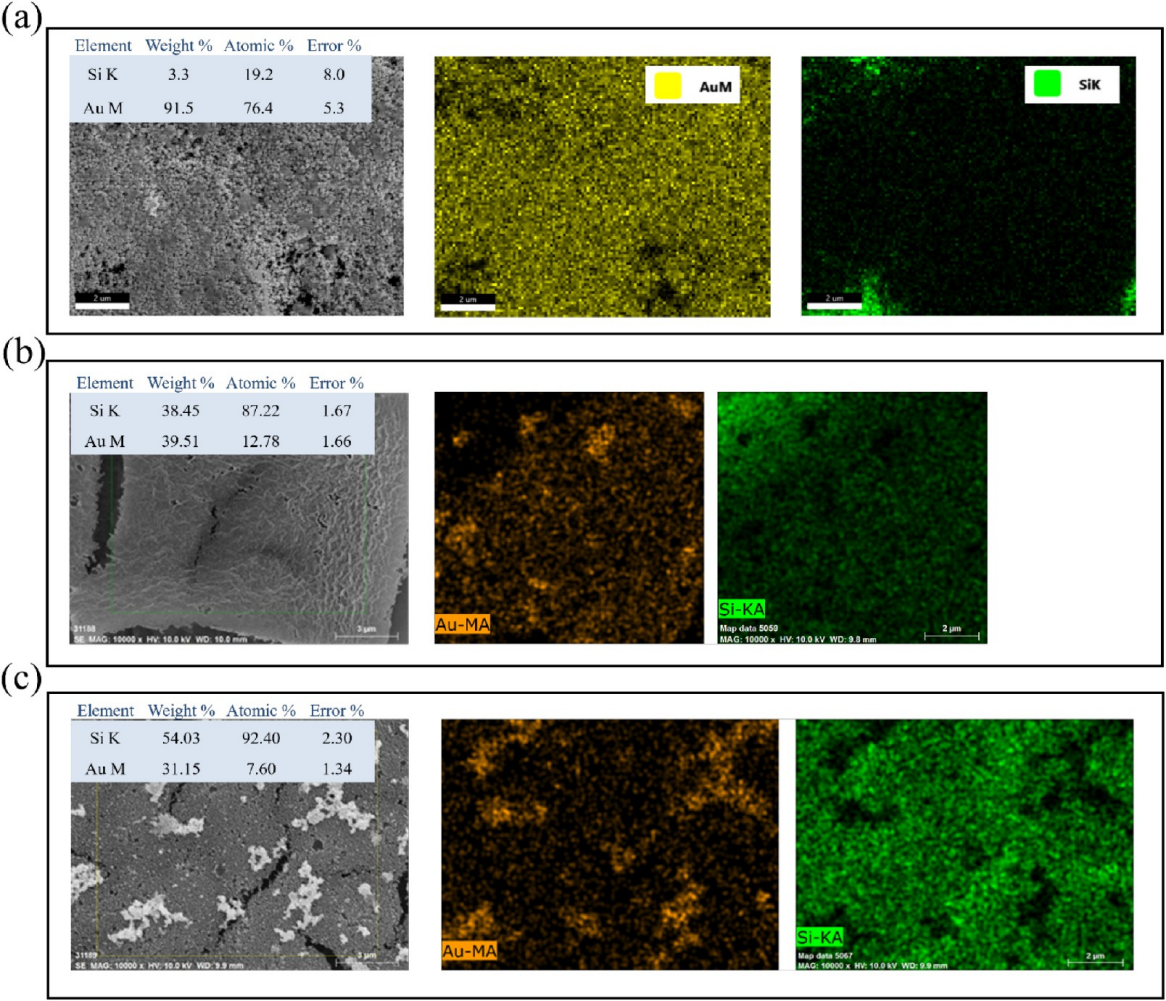
SEM–EDS elemental mapping of PSi samples prepared with 0.5 mM metal precursors after gold immersion deposition: (a) K_2_PtCl_4_-, (b) HAuCl_4_-, and (c) K_2_PdCl_4_-treated surfaces. Each panel includes SEM image, Au and Si elemental maps, and corresponding EDS quantification. The Pt-treated sample exhibits nearly complete Au coverage (∼76 at. % Au), the Au-treated sample shows partial metallization (∼13 at. % Au), and the Pd-treated sample displays sparse, clustered Au features (∼8 at. % Au).

To examine the effect of precursor concentration on gold deposition, SEM–EDS mapping was conducted on samples prepared with 5 mM metal precursors—ten times higher than in Figure 9. The results (Figure 10) show that increasing concentration does not universally enhance gold metallization and may, in some cases, be detrimental to SERS substrate formation. The K_2_PtCl_4_-pretreated sample (Figure 10a) exhibited 75.6 at. % Au and 18.5 at. % Si, nearly identical to the 0.5 mM result (Figure 9a). The Au and Si maps confirm uniform coverage and minimal exposed silicon, indicating that Pt nucleation sites formed at low concentration are already sufficient for complete gold growth. This concentration-independent behavior demonstrates that optimal SERS substrates can be achieved using minimal Pt precursor, lowering cost without compromising performance. For the HAuCl_4_-pretreated sample (Figure 10b), Au coverage increased only slightly from 12.8 to 13.2 at. %, with persistent surface heterogeneity and strong Si signal (86.8 at. %). The limited improvement suggests that Au-on-Au self-catalytic growth is not strongly influenced by precursor concentration under the present electrochemical conditions. In contrast, the K_2_PdCl_4_-pretreated sample (Figure 10c) showed a significant decrease in Au content—from 7.6 to 3.4 at. %—accompanied by dense Si signal (95.2 at. %). The surface appears dominated by Pd aggregates with isolated gold clusters, indicating that excessive Pd loading leads to surface passivation and inhibited Au nucleation. Overall, comparison between 0.5 mM and 5 mM conditions confirms that precursor concentration must be carefully optimized. Pt precursors achieve effective and stable Au growth even at low concentrations, Au precursors show limited sensitivity to concentration, and Pd precursors exhibit reduced performance at higher loadings due to aggregation. These results emphasize that controlled nanoparticle architecture and interfacial compatibility are more critical than simply increasing metal precursor concentration for achieving high-quality SERS-active bimetallic substrates.

**Figure 10:**
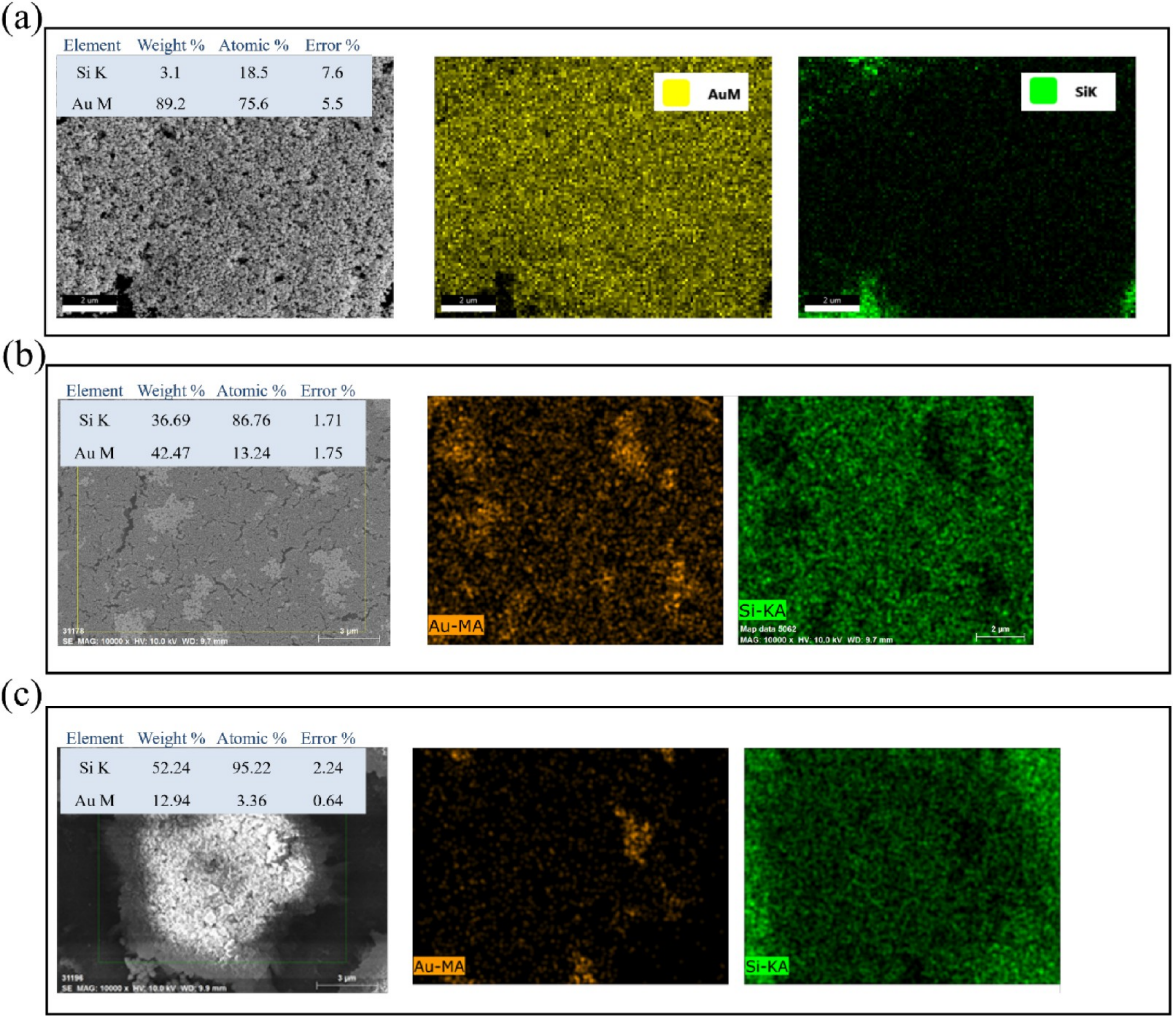
SEM–EDS elemental mapping of PSi samples prepared with 5 mM metal precursors after gold immersion deposition: (a) K_2_PtCl_4_-, (b) HAuCl_4_-, and (c) K_2_PdCl_4_-treated surfaces. Each panel includes SEM image, Au and Si maps, and quantitative EDS results. Pt-assisted samples maintain extensive Au coverage (∼75 at. % Au) independent of concentration, HAuCl_4_-treated samples show limited improvement (∼13 at. % Au), and Pd-treated samples exhibit degraded performance (∼3 at. % Au) due to excessive aggregation.

### 3.6 SERS Performance of Metal Precursor Systems at Different Concentrations

Figure 11 compares the Raman spectra of R6G obtained from SERS substrates prepared with different precursor concentrations. The HAuCl_4_-based system exhibited the strongest overall enhancement, with clearly defined R6G peaks at 612, 773, 1362, 1509, and 1573 cm^–1^.^39^ As the precursor concentration increased from 0.5 to 5 mM, signal intensity rose by approximately 15–20 times, consistent with the increased Au nanoparticle density observed in SEM and EDS analyses. Au-based substrates thus provided the highest absolute intensity and strong detection stability. However, the fluorescence background remained higher than that of Pt-based samples, limiting performance in long-integration or low-signal measurements. The K_2_PtCl_4_-based system produced slightly lower absolute intensity but exhibited notably sharper and more symmetric Raman peaks, with minimal fluorescence background and excellent reproducibility. This combination of moderate enhancement and high spectral clarity makes Pt-based substrates particularly suitable for applications requiring quantitative precision or long acquisition times. The K_2_PdCl_4_-based system generated SERS signals of moderate intensity, in some cases comparable to or slightly exceeding those of the Pt-based substrate. However, the Pd samples displayed broader peaks and stronger background noise, leading to lower effective signal-to-noise ratios. The limited dependence on precursor concentration suggests that Pd-induced enhancement is governed by less uniform nanoparticle morphology and weaker surface plasmon coupling. Au-based substrates offered the highest absolute enhancement, whereas Pt-based substrates provided the cleanest spectra and most consistent signal-to-noise performance. Pd-based substrates showed only moderate enhancement with lower reproducibility, reflecting less favorable nanoparticle uniformity. These observations reconcile the apparent discrepancies between signal intensity and detection quality across the three systems.

**Figure 11:**
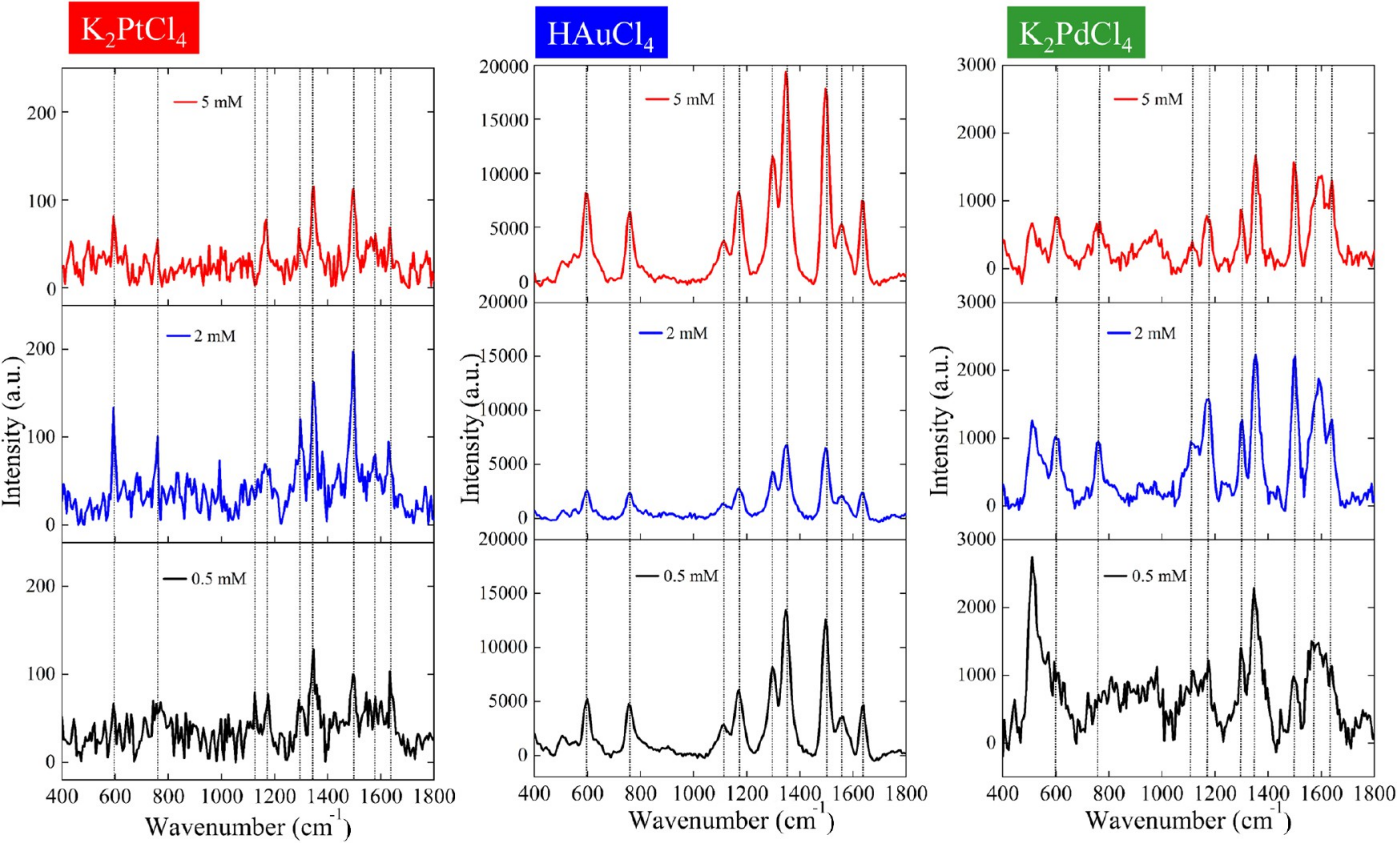
Comparison of SERS performance as a function of different noble metal precursors and their concentrations. Raman spectra of Rhodamine 6G (10^–4^ M) obtained on SERS substrates prepared with different metal precursors. Left: K_2_PtCl_4_ precursor showing SERS responses at three concentrations (0.5 mM, black; 2 mM, blue; 5 mM, red). Middle: HAuCl_4_ precursor with corresponding spectral intensity variations at the same concentration gradient. Right: K_2_PdCl_4_ precursor illustrating SERS performance at different concentrations.

### 3.7 Low-Concentration Detection Performance

To further investigate the practical detection capabilities and validate the advantage of low fluorescence background, we performed SERS measurements using a lower R6G concentration of 10^–6^ M with an extended integration time of 60 s. Figure 12 shows a direct comparison of the three substrate types under these challenging detection conditions. At this low analyte concentration, the performance differences among the substrates become dramatically evident. The K_2_PtCl_4_-treated substrate, despite showing relatively modest absolute intensity (∼200–300 au), successfully yields clearly distinguishable R6G characteristic peaks at 612, 770, 1310, 1360, 1510, and 1570 cm^–1^ against a minimal baseline. The flat background and sharp peak profiles enable reliable identification of the analyte even at this dilute concentration. In contrast, both HAuCl_4_- and K_2_PdCl_4_-treated substrates, which exhibited superior absolute intensities at 10^–4^ M concentration, fail to provide usable spectra at 10^–6^ M. The HAuCl_4_-treated sample shows a broad, intense fluorescence background (∼25000–40000 au) that completely obscures any R6G spectral features. Similarly, the K_2_PdCl_4_-treated substrate displays severe fluorescence interference (∼17000–30000 au), rendering the R6G peaks nearly indistinguishable from baseline noise. These results provide critical insights into the practical implications of fluorescence background in SERS detection. At moderate concentrations (10^–4^ M), sufficient analyte molecules are present to generate strong SERS signals that can overcome background fluorescence, resulting in comparable signal-to-noise ratios across different substrates. However, at lower concentrations (10^–6^ M), background fluorescence becomes the limiting factor. High absolute SERS intensity loses its advantage if the spectral information is masked by fluorescence, whereas low-background substrates maintain their analytical utility by preserving spectral clarity.

**Figure 12:**
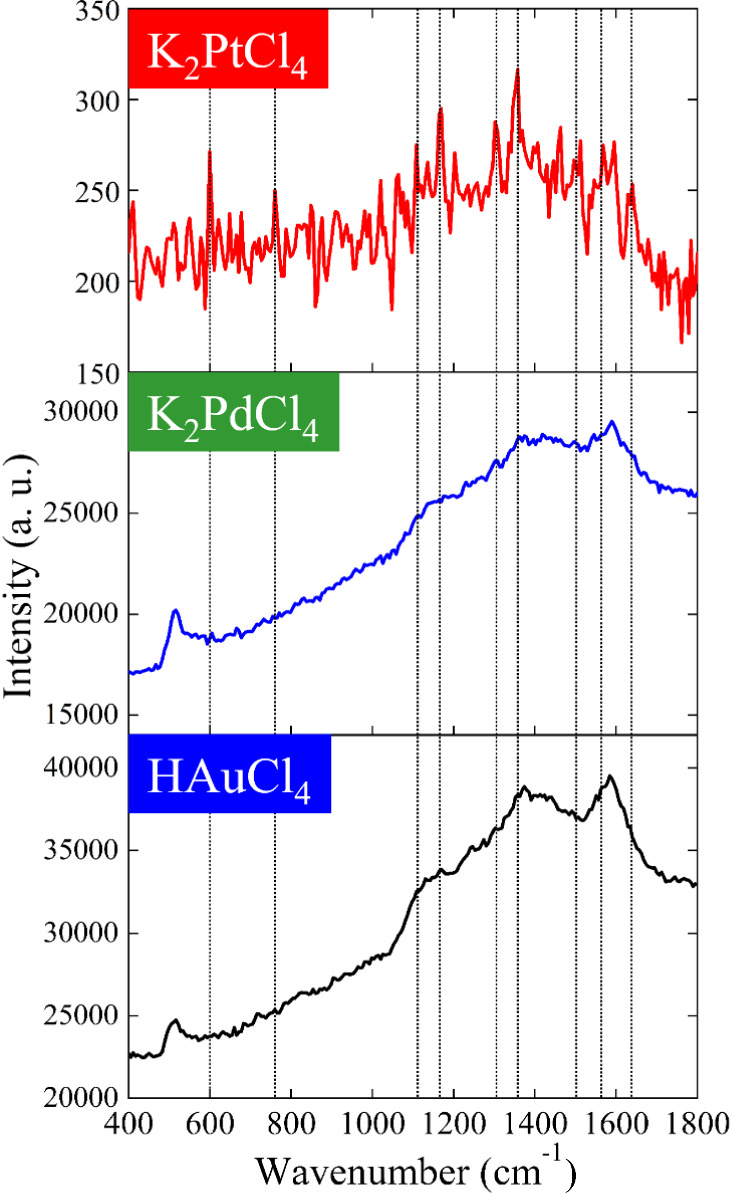
SERS spectra of 10^–6^ M R6G measured on substrates prepared with different metal precursors of 2 mM after gold deposition. The integration time of Raman measurement was 60 s.

### 3.8 Analysis of XPS Results at Different Precursor Concentrations and after Gold Deposition

Figure 13 shows the XPS spectra of substrates prepared with different precursor concentrations before and after gold deposition, highlighting the distinct surface chemical states of each metal system. In the K_2_PtCl_4_ system, the Pt 4f spectra exhibit the characteristic doublet of metallic Pt (4f_7_/_2_ ≈ 71.2 eV, 4f_5_/_2_ ≈ 74.5 eV). Increasing precursor concentration from 0.5 to 5 mM strengthens the Pt peak intensity, consistent with the higher Pt loading observed by EDS. After gold deposition, the Pt signal weakens due to partial coverage by the Au overlayer but remains visible, indicating the presence of Pt–Au composite surfaces. Slight peak-shape changes at higher concentrations suggest minor electronic interaction between Pt and Au. For the HAuCl_4_ system, the Au 4f peaks at ∼84.0 and ∼87.7 eV correspond to metallic Au both before and after deposition. The Au intensity increases with concentration up to 2–5 mM, in agreement with compositional data. Postdeposition spectra show substantially enhanced and symmetric Au 4f features, confirming additional metallic Au formation with minimal chemical-state variation. The K_2_PdCl_4_ system shows broader Pd 3d doublets (3d_5_/_2_ ≈ 335.3 eV, 3d_3_/_2_ ≈ 340.6 eV), reflecting mixed Pd oxidation states. After gold deposition, Pd peaks decrease in intensity and exhibit slight asymmetry, suggesting surface modification and partial oxidation during the process. XPS results verify that all three precursor systems retain metallic states after gold deposition, with Pt surfaces forming stable Pt–Au composites, Au systems maintaining metallic continuity, and Pd systems showing more heterogeneous chemical environments. These variations correlate with the distinct morphologies and SERS behaviors discussed earlier.

**Figure 13:**
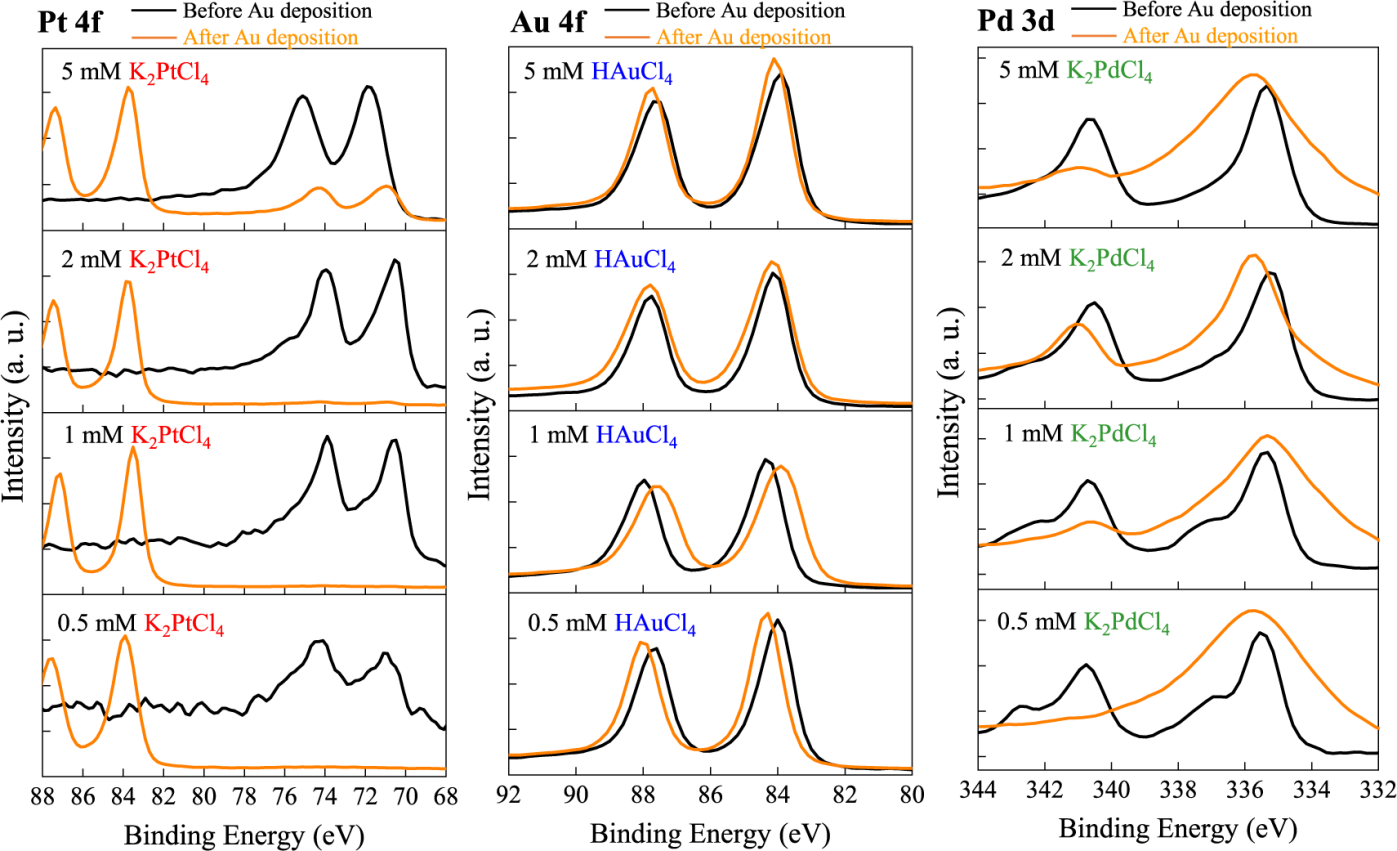
XPS analysis of substrates prepared with different concentrations of noble metal precursors before and after gold deposition.

Table 1 summarizes the quantitative results, showing that different metal precursors markedly affect the thickness and composition of the PSi layer. Under the DI-water control, the baseline thickness was ∼2.0 μm. For the K_2_PtCl_4_ system, the layer increased from 2.0 μm (0.5 mM) to 2.5 μm (2 mM) but decreased to 1.8 μm at 5 mM, indicating a nonlinear response of etching kinetics to Pt-ion concentration. The HAuCl_4_ system exhibited a monotonic decrease from 2.0 to 1.4 μm as concentration rose to 5 mM, consistent with stronger Au^3+^ reduction that suppresses Si dissolution. In contrast, the K_2_PdCl_4_ system showed minimal variation (1.8–2.5 μm), suggesting a weaker influence of Pd^2+^ on etching behavior. XPS analysis before Au immersion confirmed clear concentration-dependent metal incorporation. In the K_2_PtCl_4_ system, Pt content increased nearly linearly from 0.6 to 11.8 at. % (0.5–5 mM), indicating controllable electrochemical deposition. The HAuCl_4_ system displayed a nonmonotonic trend—Au content decreased slightly at 1 mM then rose sharply to ∼31 at. % at 5 mM—consistent with variable reduction kinetics of Au^3+^. For the K_2_PdCl_4_ system, Pd content increased steadily from 2.1 to 12.0 at. %, showing the most predictable behavior. After Au immersion, distinct differences in gold incorporation were observed. The control sample contained only ∼7.7 at. % Au, confirming limited nucleation on bare PSi. Pt-modified substrates achieved the highest Au loading, reaching ∼73 at. % at 1 mM, whereas higher Pt concentrations led to reduced Au content, reflecting surface saturation. HAuCl_4_-treated samples exhibited a more gradual rise in Au coverage (28–48 at. %), while K_2_PdCl_4_-treated samples showed irregular behavior, with high deposition at 0.5 mM (∼62 at. %) but reduced efficiency at higher levels. These quantitative results highlight distinct nucleation efficiencies among the metal systems. Pt provides the most effective promotion of Au growth at moderate concentrations, Au shows a self-catalytic but concentration-dependent effect, and Pd exhibits inconsistent nucleation behavior. The data confirm that SERS performance depends not solely on total metal loading but on the interplay between precursor type, deposition morphology, and surface structure.

**Table 1:**
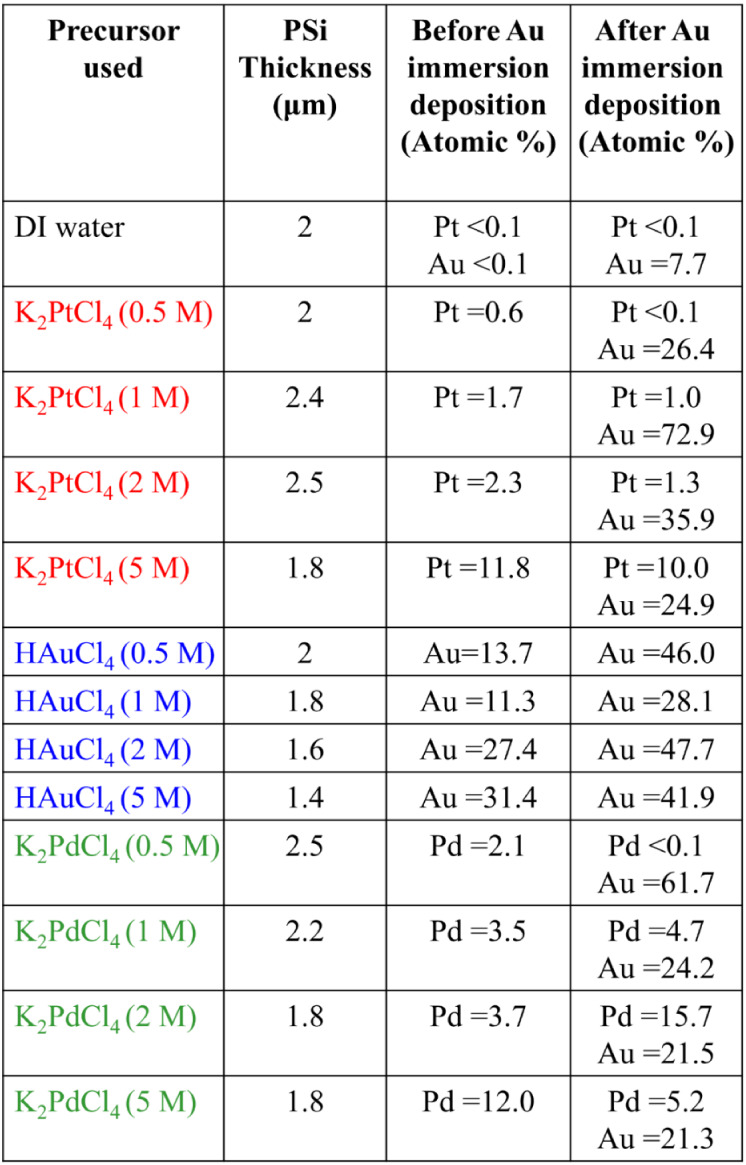
PSi Thickness and Elemental Composition before and after Au Immersion as a Function of Precursor Type and Concentration

### 3.9 SERS Substrate Reusability Performance

Figure 14 presents the SERS performance of substrates prepared with 1 mM K_2_PtCl_4_ over ten consecutive detection–cleaning cycles using Rhodamine 6G (R6G) as the probe molecule. The characteristic R6G peaks at 612, 773, 1362, 1509, and 1573 cm^–1^ were clearly observed in all cycles, confirming stable spectral reproducibility. Signal intensity gradually decreased with repeated use—remaining nearly constant for the first three cycles, then declining steadily from the fourth onward. After ten cycles, the intensity retained approximately 30–40% of its initial value, indicating that the substrates maintained partial SERS activity after multiple reuses. The postcleaning spectra (black curves) showed nearly complete removal of R6G signals after each cycle, confirming the high efficiency of the cleaning process and minimal irreversible adsorption. The cleaned spectra closely resembled those of pristine substrates, demonstrating effective regeneration between uses. The gradual signal reduction is likely related to minor mechanical wear and partial nanoparticle loss during repeated cleaning. Nevertheless, the substrates exhibited stable and reproducible performance through at least five effective detection cycles, offering clear cost and material-saving advantages over single-use SERS substrates. From a practical perspective, the combination of good reusability, reliable signal retention, and efficient regeneration highlights the structural robustness of Pt-modified PSi substrates. Their performance stability and economic feasibility make them well suited for routine sensing, teaching demonstrations, and applications where moderate detection sensitivity suffices.

**Figure 14:**
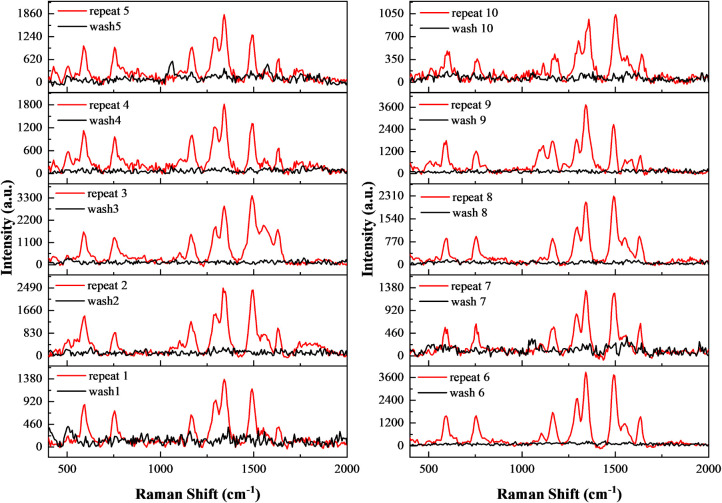
Reusability performance evaluation of SERS substrates showing 10 repeated detections of 10^–4^ M Rhodamine 6G using SERS substrates prepared with 1 mM K_2_PtCl_4_.

## 4 Conclusions

This work establishes a one-step electrochemical etching strategy enabling the simultaneous formation of porous silicon (PSi) and in situ deposition of noble metal nanoparticles, providing a direct and controllable route to fabricate bimetallic SERS substrates. Comprehensive SEM–EDS and XPS analyses confirmed that the choice of metal precursor critically determines nanoparticle distribution, nucleation efficiency, and gold metallization behavior. The systematic comparison among K_2_PtCl_4_, HAuCl_4_, and K_2_PdCl_4_ systems revealed that precursor type, rather than concentration, governs the etching dynamics and interfacial chemistry. Bimetallic interfaces were found to affect not only SERS signal intensity but also the quality and reproducibility of spectral features. K_2_PtCl_4_-treated substrates exhibited ultralow fluorescence background, sharp Raman peaks, and stable reusability—properties advantageous for high-precision and reproducible spectral analysis rather than sensitivity enhancement. HAuCl_4_-treated substrates produced the highest absolute intensities, while K_2_PdCl_4_-treated substrates showed irregular morphology and inconsistent enhancement, underscoring the importance of controlled nucleation architecture. Additional low-concentration measurements (10^–6^ M) confirmed that the low-fluorescence characteristic of Pt-based substrates becomes essential for trace-level detection. The combined morphological and chemical analyses suggest that interfacial coupling within Pt–Au and Au–Si systems may influence field uniformity and spectral fidelity. These findings outline a framework for noble-metal interface engineering, providing design guidance for SERS substrates optimized for spectral precision, reproducibility, and cost-effective reuse. Future studies should further elucidate the relationship between interfacial electronic structures and SERS enhancement mechanisms and extend this approach to other bimetallic systems for broader analytical applications.
